# *In situ* EPR spectroscopy of a bacterial membrane transporter using an expanded genetic code†

**DOI:** 10.1039/d1cc04612h

**Published:** 2021-11-18

**Authors:** Anandi Kugele, Sophie Ketter, Bjarne Silkenath, Valentin Wittmann, Benesh Joseph, Malte Drescher

**Affiliations:** Department of Chemistry and Konstanz Research School Chemical Biology (KoRS-CB), University of Konstanz, Universitätsstraße 10 78457 Konstanz Germany malte.drescher@uni-konstanz.de; Institute of Biophysics, Department of Physics & The Center for Biomolecular Magnetic Resonance (BMRZ), Goethe University Frankfurt Max-von-Laue-Str. 1 60438 Frankfurt/Main Germany joseph@biophysik.uni-frankfurt.de

## Abstract

The membrane transporter BtuB is site-directedly spin labelled on the surface of living *Escherichia coli via* Diels–Alder click chemistry of the genetically encoded amino acid SCO-l-lysine. The previously introduced photoactivatable nitroxide PaNDA prevents off-target labelling, is used for distance measurements, and the temporally shifted activation of the nitroxide allows for advanced experimental setups. This study describes significant evolution of Diels–Alder-mediated spin labelling on cellular surfaces and opens up new vistas for the the study of membrane proteins.

## Abstract 2

The membrane transporter BtuB is site-directedly spin labelled on the surface of living bacteria *via* Diels–Alder click chemistry.

## Graphical abstract

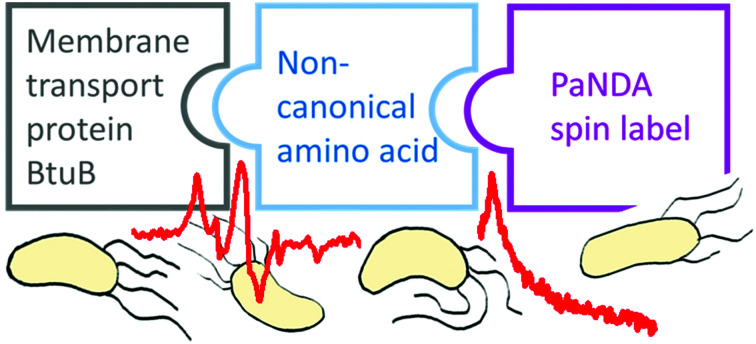

*In situ* investigation of proteins is key for comprehending the role of native environment on their structure and dynamics, but is a challenging task. To date, such studies especially on membrane proteins are underrepresented as they face many obstacles such as low expression yields and difficulty for specific labelling in the complex native membranes.^1^ To observe the protein of interest in the cellular environment spectroscopically, specific markers are required. Förster resonance energy transfer (FRET) can provide the average distance between fluorophores between rather bulky donor and acceptor fluorophores.^2^ As a complementary approach, site-directed spin labelling (SDSL) in combination with electron paramagnetic resonance (EPR) spectroscopy is a powerful biophysical tool,^3–5^ as the majority of cellular components are of diamagnetic nature, thus EPR-silent. *In vitro*, most common spin labelling approaches rely on nitroxide tags, as they are small, non-perturbing, and convenient to handle.^6,7^ In particular, their ability to report on rotational dynamics through line-shape analysis,^8,9^ and the possibility to perform distance determinations,^10^ provide unmatched spectroscopic characteristics. The best-known nitroxide spin label is the methanethiosulfonate spin label (MTSSL), which can be covalently attached to sulfhydryl groups of accessible cysteine residues in proteins.^11^ Most often genetic engineering of the protein of interest is required to eliminate undesired cysteines and, in turn, place new ones at designated sites.

In the past years, many *in vivo* EPR studies relied on the transfer of spin labelled proteins into cells, granting valuable insights into the behavior of proteins in their native environment. Beneficial reduction-stable paramagnetic centers include gadolinium,^12^ trityl^13^ or sterically shielded nitroxides.^14^ When aiming for in-cell approaches with membrane proteins, it is inevitable to perform spin labelling directly in the cellular environment. However, targeting cysteines or other native amino acids for spin labelling limits bioorthogonality, as these are ubiquitously present throughout cells. In turn, expansion of the genetic code by noncanonical amino acids (ncAA) is a promising alternative, which has proven its suitability for various biochemical and biophysical applications.^15^ For this purpose, orthogonal aminoacyl-tRNA-synthetase (aaRS)-tRNA pairs enable the selective charging of a nonsense suppressor tRNA (*e.g.* an amber codon (TAG)) with a ncAA.^16^ This technique introduces only minimal modifications into proteins and offers a wide range of highly selective reaction schemes.^17^ However, for EPR applications, the potential of ncAA-based bioorthogonal labelling is still in its infancy, especially in the context of living cells.^18^ A spin labelling scheme linking the ncAA *p*-Acetyl-l-phenylalanine to a nitroxide was pioneered in 2009,^19^ while the labelling of green fluorescent protein (GFP) by azide–alkyne cycloaddition in *Escherichia coli* (*E. coli*) represented significant advancements in this field.^20,21^ Further refinement of this technique has allowed even for distance measurements inside *E. coli* cells.^22^

Recently, we presented the first approach applying inverse electron-demand Diels–Alder click chemistry^23–27^ for SDSL of model proteins *in vitro*.^28^ The photoactivatable nitroxide for Diels–Alder (PaNDA) spin label (Fig. 1A) distinguishes itself by an *o*-nitrobenzyl-based photoremovable protecting group (PPG) for the TEMPO-based nitroxide.^29–32^ Upon UV irradiation at the desired timepoint, the PPG can efficiently release the nitroxide. The temporal control of the paramagnetic potential is expected to be especially useful to circumvent the rapid degradation of radicals in the reducing biological environment.^33^ PaNDA features a tetrazine moiety for rapid attachment to proteins without the need for potentially toxic catalysts. As a counterpart, the cyclooctene- or cyclooctyne-bearing ncAA derivatives TCO- and SCO-l-lysine are known for exceptionally high reaction rates and stability,^34^ and can be incorporated into the protein of interest by the tRNA^Pyl^/PylRS^AF^ synthetase.^35^

**Fig. 1:**
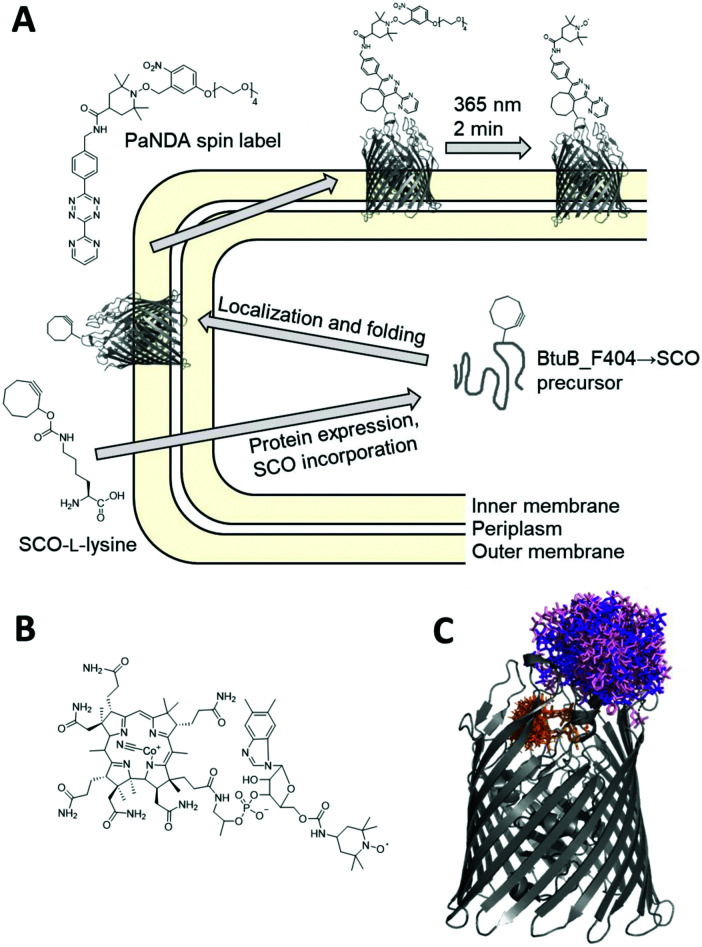
Site-directed spin labelling of a membrane transporter in intact *E. coli*. (A) Schematic overview of the site-directed spin labelling procedure using the genetically encoded ncAA SCO-l-lysine and the PaNDA spin label. (B) Chemical structure of TEMPO-CNCbl. (C) Crystal structure of BtuB (PDB entry 1NQH) with PaNDA rotamers at loop site 404. The purple and light pink shade indicate the rotamer populations for the two possible regioisomers resulting from the conjugation of PaNDA to SCO-l-lysine, as determined by MtsslWizard. TEMPO-CNCbl with its rotamers determined with MMM is drawn in orange.

Here, we report on the application of PaNDA for spin labelling and Double Electron–Electron Resonance (DEER or PELDOR) spectroscopy of the cobalamin transporter BtuB in intact *E. coli*. BtuB is responsible for the transport of vitamin B_12_ (cobalamin) into the periplasm, and has been extensively studied *in situ* using MTSSL- and trityl-based EPR spectroscopy.^36–42^ BtuB lacks native cysteines, as the majority of outer membrane proteins does. Still, certain fractions of off-target labelling have consistently been detected when labelling whole cells using MTSSL. Consequently, BtuB is perfectly suited to capitalize on the use of ncAA in combination with the PaNDA spin label (Fig. 1A).

To enable ncAA incorporation, we modified a BtuB plasmid by installing an amber codon at position 404. This loop site is known from previous studies^42^ and is located on the extracellular side. Together with a second plasmid, which encodes the tRNA^Pyl^/PylRS^AF^ pair,^35^ BtuB-deficient RK5016 *E. coli* were transformed.^43^ The cotransformation using a constitutive and an inducible vector makes our expression system unique. While the constitutive expression of BtuB_F404 → ncAA in the minimal medium enables comparably slow protein expression and controlled membrane integration, the arabinose-induced expression of the PylRS^AF^ synthetase ensures efficient ncAA incorporation. We expressed BtuB in the presence of 1 mM TCO- or SCO-l-lysine using 0.2% arabinose to induce the PylRS^AF^ overnight. Sufficient expression yields of the full-length target protein (∼66 kDa) were detected only with SCO-l-lysine (SCO; Fig. 2A and Fig. S3, ESI†) for unknown reasons, as both ncAA yielded similar incorporation yields for other proteins.^28^ When the cotransformed RK5016 *E. coli* were grown in the absence of SCO-l-lysine during expression, no BtuB was observed for the amber mutant (Fig. S4, ESI†), which confirms the integrity of the expression system. Titrating the *E. coli* cells with TEMPO-modified cobalamin^42^ (TEMPO-CNCbl; Fig. 1B) allows for semi-quantitative assessment of expression levels due to its fast binding kinetics, and revealed ∼12 μM BtuB_F404 → SCO in the cell pellet (Fig. S7, ESI;† for BtuB_F404 (wt) ∼30 μM). The line shape of X-band EPR spectra also proves, that the incorporated SCO-l-lysine does not hinder TEMPO-CNCbl binding (Fig. S5–S7, ESI†).

**Fig. 2:**
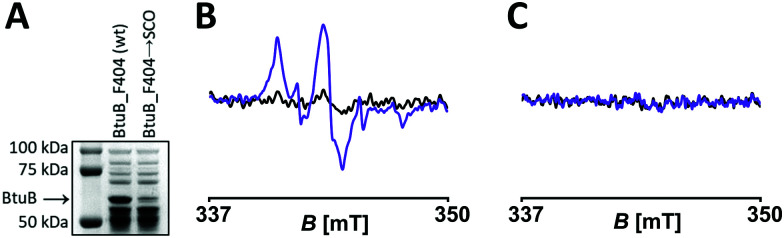
Expression and *in situ* spin labelling of BtuB variants with PaNDA. (A) 10% SDS PAGE of *E. coli* expressing indicated BtuB mutants. Experimental X-band cw EPR spectra before (black) and after (purple) irradiation of (B) BtuB_F404 → PaNDA and (C) BtuB_F404 (wt) incubated with PaNDA.

To analyze the suitability of PaNDA for *in situ* spin labelling, after expression excess ncAA was removed by adding fresh minimal medium and by repeated washing steps. The labelling reaction was performed at a cell density corresponding to OD_600_ = 15 in the presence of 150 μM PaNDA for 45 minutes. Excess label was removed by pelleting and one washing step, before cells were transferred to a micropipette. As PaNDA is EPR-active only after irradiation, UV light of 365 nm was applied, which minimizes harm to bacteria^44^ (Fig. S13, ESI†) and is able to induce cleavage of the PPG^29^ within two minutes *in vitro*^28^ and, as found here, even *in situ*. Only after irradiation, we detected the characteristic nitroxide spectrum in the BtuB_F404 → PaNDA sample (Fig. 2B). The spin concentration is ∼6 μM, which corresponds to a spin labelling and deprotection yield of ∼50% in total. Compared to previous *in vitro* experiments with PaNDA^28^ and ncAA-mediated *in vivo* spin labelling studies,^20,22^ the reduced expression and labelling yield observed here is within the expected range. Moreover, we coexpressed BtuB_F404 (wt) and the PylRS^AF^ in presence of SCO-l-lysine to check for potential off-target labelling of involved components. No attachment or labelling of PaNDA to the wildtype protein or to the *E. coli* cells was detected (Fig. 2C), which is clearly advantageous to previous experiments exploiting MTSSL labelling of BtuB.

Another beneficial feature of PaNDA is the PPG, which allows for advanced experimental schemes including temporally shifted activation of the nitroxide. To see whether this is feasible *in situ*, we expressed BtuB_F404 → SCO, performed labelling with PaNDA, and added TEMPO-CNCbl (Fig. 3). Notably, PaNDA labelling does not affect TEMPO-CNCbl binding. Complete depletion of the TEMPO-CNCbl-derived nitroxide signal was detected after ∼90 minutes. After irradiation of the cells to cleave the PPG, indeed the PaNDA-derived nitroxide signal was detected. We hypothesize, that the reason for the different decay rates is the different accessibility of the labels towards reducing agents. Altogether, this proves that the PPG is stable for at least three hours (including the labelling and sample preparation time) under *in situ* conditions.

**Fig. 3:**
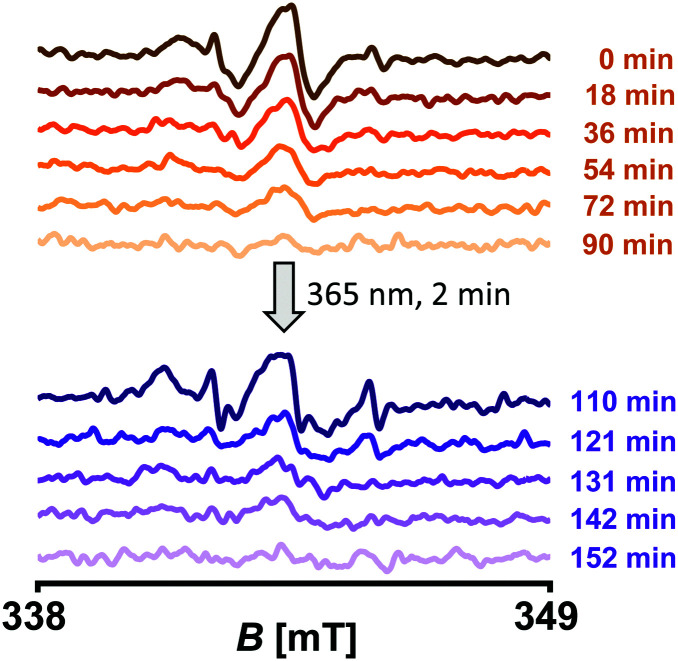
Temporal control of the paramagnetic potential of the PaNDA spin label *in situ*. *E. coli* expressing BtuB_F404 → SCO were spin labelled with PaNDA, mixed with TEMPO-CNCbl (which corresponds to the indicated timepoint 0 min), and cw EPR spectra were recorded (orange shades; spectra were averaged over 12 scans). When the TEMPO-CNCbl derived signal had vanished, cells were irradiated and cw EPR spectra were recorded to measure the PaNDA-derived signal (purple shades; spectra were averaged over 8 scans).

Previously, distances in BtuB were determined either between two spin labelled cysteine residues, or in combination with TEMPO-CNCbl.^36–42^ As reference, we expressed BtuB_F404C and spin labelled with MTSSL in the isolated outer membranes (OM) and *in situ* resulting in the side chain BtuB_F404 → R1. After addition of TEMPO-CNCbl 4-pulse DEER experiments were performed (Fig. S14 and S15, ESI†). The resulting modulation depths are Δ_*in situ*_ = 7.4% and Δ_OM_ = 2.7% and the maximum of the distance distributions overlays with the Multiscale Modeling of Macromolecules (MMM)^45^ simulation.

To perform EPR distance determination involving PaNDA, we produced BtuB_F404 → PaNDA both *in situ* and in the OM and added TEMPO-CNCbl. After irradiation, samples containing 20% d8-glycerol were frozen, and DEER was measured (Fig. 4 and Fig. S9–S11, ESI†). By doing this, we combined four challenging aspects in one experiment for the first time: (i) a membrane transporter as protein of interest (which suffer in general from low expression and challenging EPR spectroscopy), (ii) the use of ncAA, (iii) *in situ* spin labelling and (iv) *in situ* DEER measurement. Especially, low expression of the ncAA-containing BtuB reduces the protein concentration (Fig. 2A), resulting in low overall spin concentration as well as lower signal-to-noise ratio and short length of the obtained DEER trace. This leaves room for improvements in future experiments. However, especially with regard to modulation depths (Δ_*in situ*_ = 9.5% and Δ_OM_ = 3.0%), the data we acquired confirm an adequate labelling degree and the general suitability of our approach for DEER measurements.

**Fig. 4:**
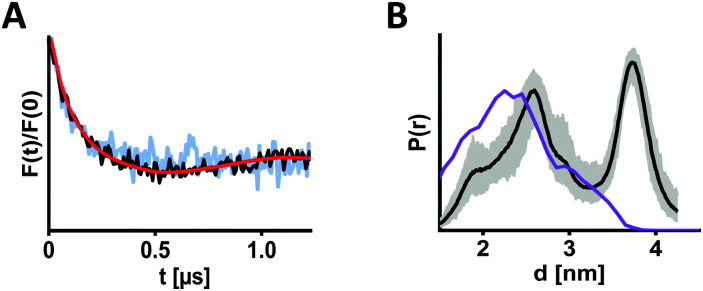
DEER distance measurements of BtuB using Diels–Alder click chemistry. (A) BtuB_F404 → SCO was spin labelled with PaNDA *in situ* (black) or in isolated outer membranes (light blue; for full length trace see Fig. S11, ESI†), and DEER was measured after irradiation in presence of TEMPO-CNCbl. For comparison, form factors after background subtraction were scaled with respect to the modulation depth (Δ_*in situ*_ = 9.5% and Δ_OM_ = 3.0%). (B) Corresponding *in situ* distance distribution (black) with validation (grey area). The purple line indicates the simulated distance distribution (using MtsslWizard), assuming a 1 : 1 ratio of the two possible linker regioisomers.

The DEER data for distance determination between BtuB_F404 → PaNDA and TEMPO-CNCbl looks similar *in situ* and in the OM (Fig. 4A). The corresponding distance distribution exhibits two maxima (*d*_1_ = 2.6 nm and *d*_2_ = 3.7 nm), and is relatively broad in the accessible range (Fig. 4B). The width of the distribution is expected due to the linker size resulting from the combination of the relatively long lysine-based ncAA and the PaNDA spin label. To further assess the extracted distance distribution, rotamers for the PaNDA-derived linkers were generated using the MtsslWizard^46^ software (Fig. 1C and Fig. S8, ESI†). They reveal that the first part of the experimental distance distribution overlays with the simulation, while the longer distance is not described (Fig. 4B). Previous experimental^37^ and computational^47^ findings however suggested high flexibility of the extracellular loops of BtuB. As we could not reproduce this by MTSSL-labelling (Fig. S14 and S15, ESI†), we suspect the PaNDA label to induce different loop conformations.

Moreover, we spin labelled BtuB_F404 → SCO with PaNDA, left out TEMPO-CNCbl, and measured DEER *in situ*. The resulting data indicates the homogenous distribution of BtuB on the *E. coli* surface (Fig. S12, ESI†).

In summary, SCO-l-lysine was incorporated into the membrane transporter BtuB in high yields, and the surface of living *E. coli* provides a suitable environment for PaNDA spin labelling as well as nitroxide activation *via* irradiation and spontaneous oxidation. The spin labelling and deprotection conditions developed for *in situ* EPR are completely biocompatible, and allowed for a DEER experiment involving the PaNDA label directly on the surface of *E. coli*.

This study provides the first spin labelling scheme for membrane proteins using an expanded genetic code, and the first application of Diels–Alder chemistry for spin labelling of proteins in the cellular environment.

This project has received funding from the European Research Council (ERC) under the European Union's Horizon 2020 research and innovation programme (Grant Agreement number: 772027 – SPICE – ERC-2017-COG). B. J. thanks DFG for the financial support through the Emmy Noether Program (JO 1428/1-1). We thank Frederike Krasucki for experimental contributions.

## Conflicts of interest

There are no conflicts to declare.

## Supplementary Material

CC-057-D1CC04612H-s001
