# Supplementary material for: In situ EPR spectroscopy of a bacterial membrane transporter using an expanded genetic code
Source: Chem Commun (Camb). 2021 Nov 18;57(96):12980–3. doi: 10.1039/d1cc04612h (PMC8640571; doi:10.1039/d1cc04612h)
Supplement: CC-057-D1CC04612H-s001 [file CC-057-D1CC04612H-s001.pdf]

Electronic Supplementary Material (ESI) for ChemComm.
This journal is © The Royal Society of Chemistry 2021

Electronic Supplementary Information for "*In situ* EPR Spectroscopy of a Bacterial Membrane Transporter using an Expanded Genetic Code"

Anandi Kugele, Sophie Ketter, Bjarne Silkenath, Valentin Wittmann, Benesh Joseph* and Malte Drescher*

Table of Contents

Experimental Procedures **2**

Preparation of chemically competent RK5016 *E. coli* 2

Experiments with the cysteine mutant BtuB_F404C 2

Mutagenesis of a BtuB amber codon variant 2

Transformation of *E. coli* 2

Expression of BtuB_F404 (wt) and BtuB_F404→ncAA 3

SDS-PAGE 3

Incubation of BtuB with TEMPO-CNCbl 4

Spin labelling of BtuB with PaNDA 4

Cw EPR measurements 4

Pulsed EPR spectroscopy 5

Generation of PaNDA rotamers and simulation of the distance distribution 5

Supplementary Figures **6**

Figure S1: Chemical structures of compounds used 6

Figure S2: BtuB amino acid sequence 7

Figure S3: Expression levels of BtuB variants analyzed by SDS-PAGE 8

Figure S4: Analysis of synthetase integrity by SDS-PAGE 8

Figure S5: Comparison of expression levels of BtuB variants 9

Figure S6: Optimization of BtuB_F404→SCO expression levels 10

Figure S7: Titration of BtuB_F404→SCO with TEMPO-CNCbl 10

Figure S8: Generation of PaNDA rotamers 11

Figure S9: DEER data of PaNDA- and TEMPO-CNCbl-labelled sample *in situ* 12

Figure S10: PaNDA labelling in OM 13

Figure S11: DEER data of PaNDA- and TEMPO-CNCbl-labelled sample in OM 14

Figure S12: DEER data of PaNDA-labelled sample 15

Figure S13: Viability of *E. coli* 15

Figure S14: DEER data of MTSSL- and TEMPO-CNCbl-labelled sample *in situ* 16

Figure S15: DEER data of MTSSL- and TEMPO-CNCbl-labelled sample in OM 17

Supplementary References **18**

Experimental Procedures

Preparation of chemically competent RK5016 *E. coli*

5 mL of LB medium were inoculated with 5 µL chemically competent RK5016[1] *Escherichia coli* (*E. coli*) cells and grown overnight at 37 °C under vigorous shaking at 180 rpm. 200 µL of the overnight culture were used to inoculate 50 mL of fresh LB medium, and grown at 37 °C until reaching an optical density of OD_{600} = 0.5 - 0.6. The cell suspension was kept on ice for 10 min, before cells were pelleted by centrifugation for 10 min at 4.000 x g. The supernatant was discarded, and the pellet was resuspended in 50 mL of ice-cold $CaCl_2$ solution (0.1 M in pure water, filtersterilized). Cells were incubated on ice for 20 min, before the centrifugation step was repeated and the supernatant was discarded. Finally, cells were resuspended in 2.5 mL of $CaCl_2$ solution containing 10 % of glycerol (filtersterilized or autoclaved). Cells were aliquoted in 25 µL and flash frozen in liquid nitrogen, before being stored at -80 °C.

Experiments with the cysteine mutant BtuB_F404C

Expression of the cysteine variant BtuB_F404C, the isolation of bacterial membranes, MTSSL spin labelling and DEER measurements were performed as described by Joseph et al.[2, 3] DEER data (**Figure S14** and **S15**) were processed using MATLAB R2019b and the DeerAnalysis2019[4] software. The distance distributions were determined by artificial neuronal network analysis (DEERNet)[5] implemented in DeerAnalysis.

Mutagenesis of a BtuB amber codon variant

The sequence of BtuB_F404 (wt) is shown in **Figure S2**. The amber mutation (TAG) was introduced into the wildtype plasmid pAG1_BtuB_WT at amino acid position 404 as described before[2] by using an Agilent Technologies (Santa Clara, CA) QuikChange Site Directed Mutagenesis Kit. The resulting plasmid is referred to as "pAG1_BtuB_F404TAG". The correctness of all used plasmids was confirmed by sequencing.

Transformation of *E. coli*

For the experiments with BtuB wildtype (BtuB_F404 (wt)) or amber mutant BtuB (BtuB_F404→ncAA), the plasmid pEVOL_PylRS_AF (kindly gifted by Daniel Summerer) together with either pAG1_BtuB_WT or pAG1_BtuB_F404TAG were used. The respective plasmids were co-transformed into chemically competent RK5015 *E. coli* (prepared as described above). For this purpose, the reaction tube containing 25 µL *E. coli* and each 1 µL of the particular plasmids (in concentrations in the range of 125 – 500 ng/µL) was mixed by flicking and incubated 30 min on ice. The cells were then heat shocked at 42 °C for 37 seconds and incubated for another 2 min on ice, before being added to 1 mL pre-warmed (37 °C) Super Optimal Broth with catabolite repression (SOC-medium). The cells were then incubated for 1 h at 37 °C and 800 rpm, before centrifuged at

1.500 x g for 2 min. The whole cell pellet was spread on a sterile LB-Agar plate (Lennox; ROTH) containing 34 µg/mL chloramphenicol (ROTH) and 50 µg/mL carbenicillin (ROTH) and incubated overnight at 37 °C. The next day, several colonies were found and indicated successful cotransformation of the two plasmids. For long-time storage of transformed *E. coli* glycerol stocks were frozen at -80 °C.

Expression of BtuB_F404 (wt) and BtuB_F404→ncAA

The mutant BtuB_F404→ncAA contains one of the noncanonical amino acids SCO-L-lysine or TCO-L-lysine (short names: SCO and TCO; **Figure S1A and B**; both purchased from Sirius Fine Chemicals) at amino acid residue 404 instead of phenylalanine. All steps, unless otherwise stated, were performed in minimal medium (prepared as described by Joseph et al.[3]), which additionally contained 34 µg/mL chloramphenicol and 50 µg/mL carbenicillin. The MOPS buffer contained 5.2 g of MOPS and 1.6 g of NaCl per 500 mL MilliQ-water at a pH of 7.5.[3]
Co-transformed RK5016 *E. coli* with the respective plasmid(s) (see above) were used to express BtuB_F404 (wt) or BtuB_F404→ncAA as follows. For overnight cultures, 5 mL LB-medium (Lennox, ROTH) were inoculated with one colony from the agar plate overnight at 180 rpm and 37 °C. For experiments with RK5016 cells, that were not transformed with plasmids (**Figure S3 and S5**), the overnight culture was incubated with one cell aliquot (25 µL) instead of agar colonies. The next day, 1.25 mL of this overnight culture were used to inoculate 25 mL of minimal medium. The preculture was incubated for 5 hours at 33 °C and 140 rpm. Then, typically 10 mL of this preculture were used to inoculate 40 mL of fresh minimal medium. Directly before expression started, the ncAA were freshly prepared by dissolving them in 0.1 M NaOH in concentrations of 60 mM or 80 mM for SCO-L-lysine or TCO-L-lysine, respectively. Protein expression was induced with 0.2 % L-(+)-arabinose (ROTH; from a 20 % w/v stock solution in MilliQ-water, filtersterilized) in presence of 1 mM ncAA (for optimized conditions see **Figure S6**). Cells were grown overnight (for approx. 18 hours) at 33 °C and 140 rpm. The next day cells were harvested by centrifugation (4 °C, 4.000 rpm, 10 min).
To get rid of excess ncAA, the supernatant was discarded, and the pellet was resuspended by pipetting 25 x up and down in 40 mL of fresh minimal medium containing antibiotics and 0.2 % arabinose. The cells were then incubated for one more hour at 33 °C and 140 rpm in fresh Erlenmeyer flasks. After centrifugation, cells were transferred into 15 mL falcon tubes on ice and thoroughly resuspended with 15 mL MOPS buffer. Centrifugation and resuspension were repeated two more times.
The cells were finally collected in 15 mL MOPS buffer and kept on ice until labelling or use for SDS-PAGE. To determine the cell density, aliquots were further diluted to measure within the linear range (OD_{600} = 0.01 – 0.4), and the OD_{600} was measured in 1 mL plastic cuvettes using MOPS-buffer as a background reference.

SDS-PAGE

BtuB expression and ncAA integration were confirmed by SDS-PAGE of whole cell lysate (**Figure 2A,** main text, **S3 and S4**). An appropriate amount of *E. coli* cells was prepared by pelleting approx. 4 x 10^8 cells (corresponding to 100 µL cell suspension at OD_{600} = 5) at 15.000 x g for 3 min. The pellet was resuspended in 20 µL of MilliQ-water and 4 µL of 6x Laemmli loading buffer. The mixture was cooked at 95 °C for 40 min. After short centrifugation to collect the sample, 10 µL were applied to a 10 % SDS-gel. Additionally, 5 µL of a BIO-RAD Precision Plus ProteinTM Dual Color Standard were applied as marker. Gels were run at 90 V in a BIO-RAD Mini-PROTEAN Tetra System. After Coomassie Blue staining (Brillant Blau R 250, ROTH), gels were imaged using a BIO-RAD ChemiDocTM Imaging System.

Incubation of BtuB with TEMPO-CNCbl

TEMPO-CNCbl (**Figure S1C**) was synthesized as described by Joseph et al.[6] In this study TEMPO-CNCbl was used to assess the amount of expressed BtuB by titration (**Figure S5 - S7**), and for determination of inter-spin distances. For these purposes a 200 µM stock solution of TEMPO-CNCbl in MilliQ-water in an amber coloured tube was frozen at -80 °C for long-time storage. For evaluation of BtuB expression, an appropriate amount of cells (typically corresponding to 1 mL of cells with a density of OD_{600} = 5 or 15) was pelleted at 4000 rpm, 4 °C for 7 min. The volume of the pellet was made up to 25 µL with precooled MOPS buffer, before adding the indicated amount of TEMPO-CNCbl. Samples were immediately used for EPR measurements.

Spin labelling of BtuB with PaNDA

An appropriate amount of cells was pelleted at 4000 rpm, 4 °C for 7 min to yield typically 1 mL of cells with a density of OD_{600} = 15 (adequate for one CW-EPR sample), when vigorously resuspending 25 x in precooled PBS pH 7.4 buffer in amber coloured tubes (Eppendorf). For one DEER sample, the threefold amount of cells were required. 150 µM of PaNDA label (**Figure S1D**) was added (from a 10 mM stock solution in DMSO, stored at -20 °C), and labelling was performed for 45 min at 25 °C and 850 rpm in an Eppendorf ThermoMixer C. After centrifugation, cells were resuspended with 1 mL PBS buffer to remove excess label, and centrifuged again. The resulting pellet was made up with PBS buffer to a volume of 25 µL. The sample was filled into a glass capillary (HIRSCHMANN ringcaps; inner diameter 1.02 mm). For activation of the nitroxide, the capillary was placed on a table, and covered with a handheld UV lamp (UVLM-28 EL Series UV Lamp, 8 Watt, 302/365 nm, analytikjena). This lamp can be switched between two separated light sources and operates at only one of the two wavelengths at a time. Samples were irradiated for 2 min at 365 nm, and immediately used for room-temperature cw EPR measurements (**Figure 2B and C,** main text). Viability of *E. coli* cells was determined (**Figure S13**). For DEER, 48 µL of cells were retransferred to a microcentrifuge tube, mixed with an appropriate amount of TEMPO-CNCbl and 12 µL of d_8-glycerol, transferred to a 3 mm outer diameter quartz tube (Fused quartz tubing, Technical Glass Products), and immediately shock-frozen in liquid nitrogen.

Cw EPR measurements

Continuous wave (cw) EPR spectra were recorded at a BRUKER EMXnano X-band continuous wave EPR spectrometer without temperature regulation at room temperature (approx. 20 °C). A typical sample volume of 25 µL was filled into a glass capillary (HIRSCHMANN ringcaps; inner diameter 1.02 mm) and sealed with tube sealing compound (Kimble Cha-Seal) on one end. Spectra were recorded with a modulation amplitude of 1.5 G, microwave attenuation 20 dB, and a sweep width of 180 G around the center field of 3440 G. Several scans of 27 seconds scan time each were accumulated to optimize the signal-to-noise ratio (SNR). Quantitative spin concentrations of samples were obtained with the built-in EMXnano reference-free spin counting module (Xenon software, Bruker). The PaNDA labelling efficiency was estimated as the ratio of the PaNDA-derived spin concentration to the concentration of bound TEMPO-CNCbl. Spectra were processed with MATLAB R2019b (The MatWorks, Natick, USA) and plotted with Origin 2018 (OriginLab Corporation, Northampton, USA).

Pulsed EPR spectroscopy

DEER distance measurements of the PaNDA-labelled samples (**Figure 4**, main text, and **Figures S9**, **S11** and **S12**) were performed in Q-band (34 GHz) using an Elexsys E580 spectrometer (Bruker Biospin). This spectrometer operates with a SpinJet-AWG unit (Bruker Biospin) and a 150 W pulsed traveling-wave tube (TWT) amplifier (Applied Systems Engineering). The sample was held at 50 K with the EPR Flexline helium recirculation system (CE-FLEX-4K-0110, Bruker Biospin, ColdEdge Technologies) comprising a cold head (expander, SRDK-408D2) and a F-70H compressor (SHI cryogenics), controlled by an Oxford Instruments Mercury ITC. A commercial Q-band resonator (ER5106QT-2, Bruker Biospin) was used. A frequency offset of minus 80 MHz (resulting in 33.92 GHz) was chosen for the observer pulses, and a shot repetition rate of 4 ms was utilized to avoid nitroxide saturation. The length of the Gaussian shaped observer pulse was set to $\pi_{observer}$ = 36 ns, and the symmetric hyperbolic secant HS[1,1] pump pulse[7] had a fixed length of π_{pump} = 100 ns. For phase-cycling, the eight-step phase cycle [x](x)x_px as proposed by Tait and Stoll[8] was employed. The PaNDA- and TEMPO-CNCbl-labelled samples (**Figure S9** and **S11**) were recorded for 76 h. The data were processed using MATLAB R2019b and the DeerAnalysis2019[4] software, and the distance distribution was determined by artificial neuronal network analysis (DEERNet)[5] implemented in DeerAnalysis. The background sample labelled only with PaNDA (**Figure S12**) was measured for 22 h.

Generation of PaNDA rotamers and simulation of the distance distribution

Rotamers of the PaNDA spin label were calculated by using the PyMOL plugin MtsslWizard[9] to simulate spin labelling and distance distributions. For this purpose, the manual by Gregor Hagelueken and Dinar Abdullin was followed (manual for mtsslSuite, Version 2.0, January 2017, chapter 2.2 "Adding new spin labels"). Briefly, the structures of the two labelled amino acid regioisomers (featuring an aldehyde group instead of the acid group) were drawn in ChemDraw Professional 16.0 (**Figure S8A**) and fed into Chem3D 16.0. Energy of the linker was minimized by using a RMS Gradient of 0.01. The resulting PDB file was further edited in PyMOL (The PyMOL Molecular Graphics System, Version 2.4.1 Schrödinger, LLC.)[10] by removing all hydrogen atoms. All atom names of the remaining 51 atoms were individually annotated, conforming to the commonly used annotation pattern (**Figure S8B**). The 13 bonds that contribute to the rotation string were defined (**Figure S8A**), and correspondingly a new label description file was generated. The resulting PDB file and description file were fed into the respective MtsslWizard folder.

PaNDA rotamer calculations at site 404 in MtsslWizard resulted in 71 or 72 rotamers for regioisomers (1) and (2), respectively (**Figure S8C**). To predict the distance distribution between TEMPO-CNCbl and PaNDA, 24 TEMPO-CNCbl rotamers[6] were predetermined in MMM[10], exported to PyMOL and extracted to object. The final simulation of the distance distribution between these two rotamer populations was determined following the standard MtsslWizard procedure and is shown in **Figure 4B**, main text, and **Figure S9C** and **S11C**.

Supplementary Figures

Figure S1: Chemical structures of compounds used

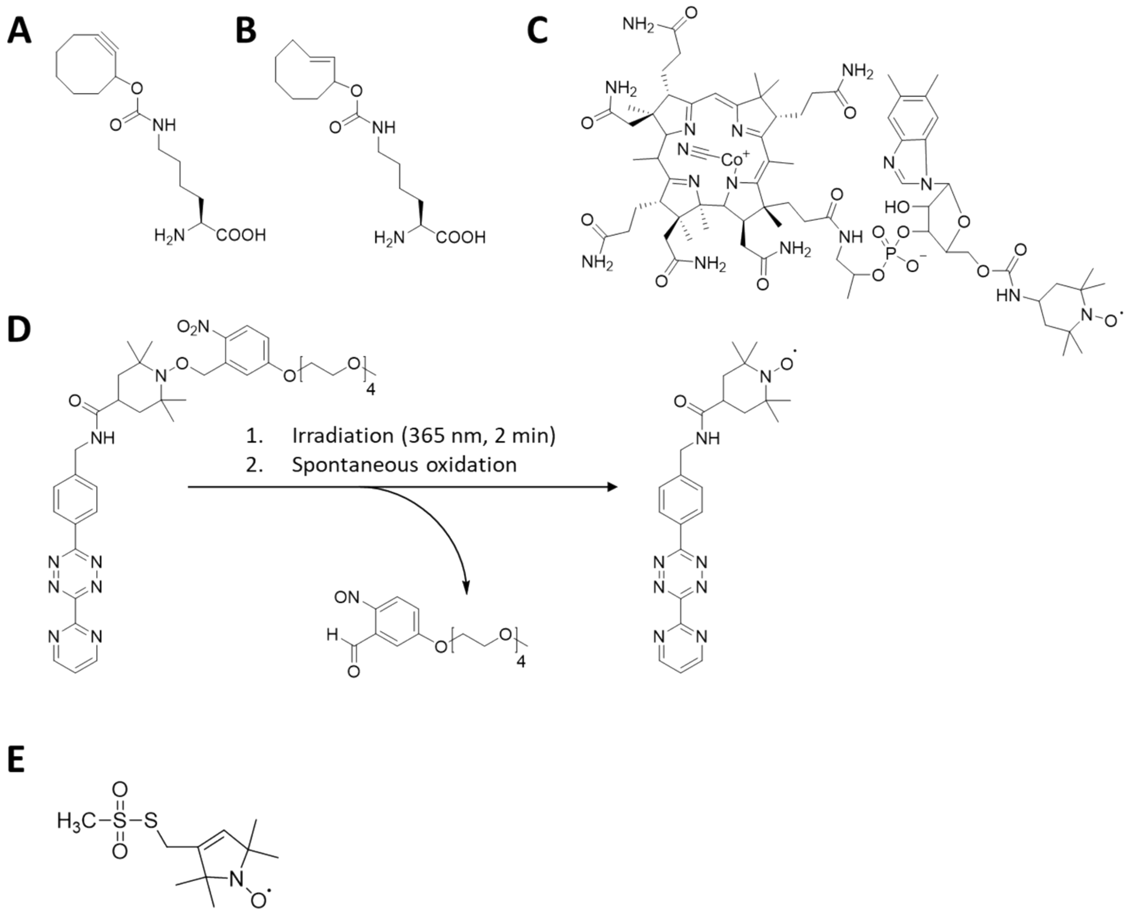

A: SCO-L-lysine

B: TCO-L-lysine

C: TEMPO-Cobalamin (TEMPO-CNCbl)

D: PaNDA spin label before (left) and after (right) activation. The structures of PaNDA bound to SCO-L-lysine are shown in **Figure S8**.

E: MTSSL spin label

Figure S2: BtuB amino acid sequence

```
        10         20         30         40         50
MIKKASLLTA CSVTAFSAWA QDTSPDTLVV TANRFEQPRS TVLAPTTVVT

        60         70         80         90        100
RQDIDRWQST SVNDVLRRLP GVDITQNGGS GQLSSIFIRG TNASHVLVLI

       110        120        130        140        150
DGVRLNLAGV SGSADLSQFP IALVQRVEYI RGPRSAVYGS DAIGGVVNII

       160        170        180        190        200
TTRDEPGTEI SAGWGSNSYQ NYDVSTQQQL GDKTRVTLLG DYAHTHGYDV

       210        220        230        240        250
VAYGNTGTQA QTDNDGFLSK TLYGALEHNF TDAWSGFVRG YGYDNRTNYD

       260        270        280        290        300
AYYSPGSPLL DTRKLYSQSW DAGLRYNGEL IKSQLITSYS HSKDYNYDPH

       310        320        330        340        350
YGRYDSSATL DEMKQYTVQW ANNVIVGHGS IGAGVDWQKQ TTTPGTGYVE

       360        370        380        390        400
DGYDQRNTGI YLTGLQQVGD FTFEGAARSD DNSQFGRHGT WQTSAGWEFI

       410        420        430        440        450
EGYRFIASYG TSYKAPNLGQ LYGFYGNPNL DPEKSKQWEG AFEGLTAGVN

       460        470        480        490        500
WRISGYRNDV SDLIDYDDHT LKYYNEGKAR IKGVEATANF DTGPLTHTVS

       510        520        530        540        550
YDYVDARNAI TDTPLLRRAK QQVKYQLDWQ LYDFDWGITY QYLGTRYDKD

       560        570        580        590        600
YSSYPYQTVK MGGVSLWDLA VAYPVTSHLT VRGKIANLFD KDYETVYGYQ

       610
TAGREYTLSG SYTF
```

Primary amino acid sequence of BtuB wildtype (BtuB_F404 (wt); FASTA sequence P06129 (BTUB_ECOLI) was accessed at www.uniprot.org). Position 404 (marked in yellow) was mutated to cysteine (BtuB_F404C) or to a noncanonical amino acid (BtuB_F404→ncAA) in this study. Note that the first 20 amino acids represent a signal peptide (marked in green), which is cleaved once the protein has localized to the membrane. Thus, the position 404 of the final protein is annotated as 424 in this representation.

Figure S3: Expression levels of BtuB variants analyzed by SDS-PAGE

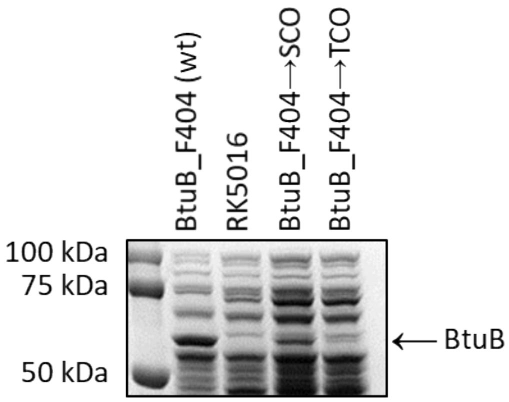

Analysis of BtuB expression levels by 10 % SDS PAGE of whole cell lysate. Cells expressed BtuB_F404 (wt, first column) or BtuB_F404→ncAA (third and fourth column). In the second column non-transformed RK5016 cells were analyzed to exclude expression of native BtuB. The arrow indicates the position of BtuB on the gel. Expression levels were significantly higher for BtuB_F404→SCO than for BtuB_F404→TCO. The corresponding cw EPR spectra after addition of TEMPO-CNCbl are shown in **Figure S5**.

Figure S4: Analysis of synthetase integrity by SDS-PAGE

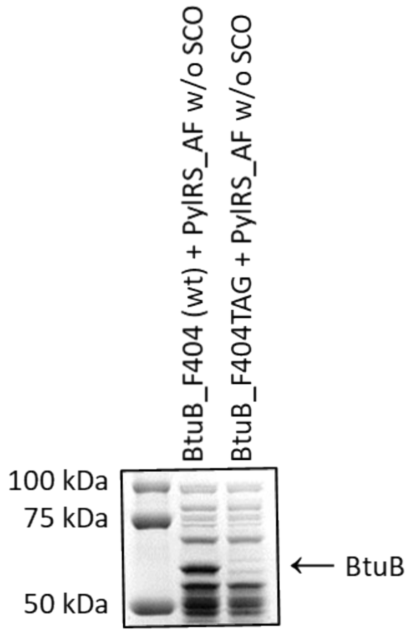

Analysis of BtuB expression levels by 10 % SDS PAGE of whole cell lysate. Cells were cotransformed with wildtype or amber mutant plasmid in addition to the PylRS^{AF} synthetase, and cultivated in medium without SCO-L-lysine. The arrow indicates the position of BtuB on the gel. Expression was found only for BtuB_F404 (wt), as it is not dependent on ncAA incorporation. Absence of BtuB in the second column suggests that no native amino acid was misincorporated in response to the amber codon. Consequently, no full-length BtuB was found.

Figure S5: Comparison of expression levels of BtuB variants

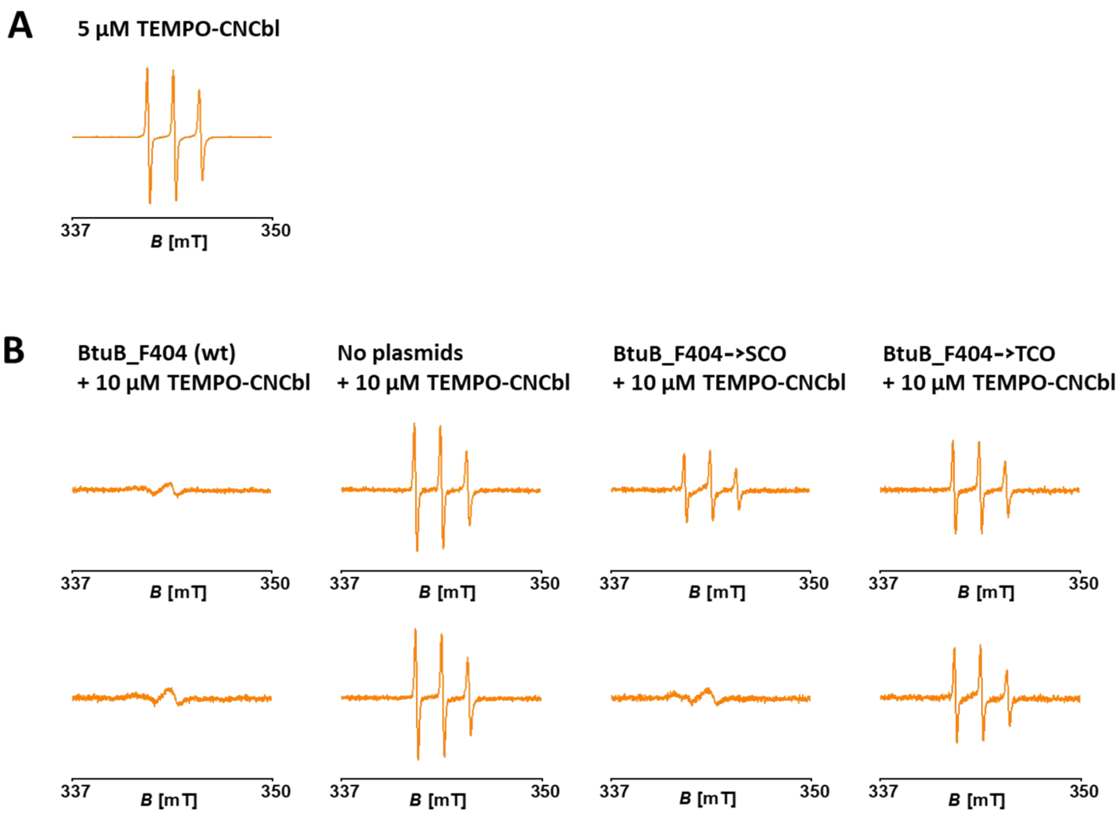

Comparison of BtuB expression levels of BtuB variants. Cw EPR spectra of (A) unbound TEMPO-CNCbl in PBS buffer and (B) TEMPO-CNCbl added to RK5016 cells expressing the indicated BtuB variants. All spectra were recorded with the same number of accumulated scans and thus scaled to the same SNR. 1 mL of *E. coli* at OD_{600} = 5 (upper line) or OD_{600} = 15 (lower line) were pelleted, resuspended with MOPS buffer in a total volume of 25 µL and mixed with 10 µM TEMPO-CNCbl. Bound TEMPO-CNCbl as observed by line broadening indicates expression of BtuB, and conforms with the BtuB expression levels detected by SDS-PAGE (**Figure S3**). While the broadened lineshape for higher concentrated cells (OD_{600} = 15, lower line) indicates successful expression of BtuB_F404⟶SCO, this effect is not observable for BtuB_F404⟶TCO. For the spectra named "No plasmids", RK5016 cells were not transformed with any plasmid, thus absence of native BtuB was found.

Figure S6: Optimization of BtuB_F404⟶SCO expression levels

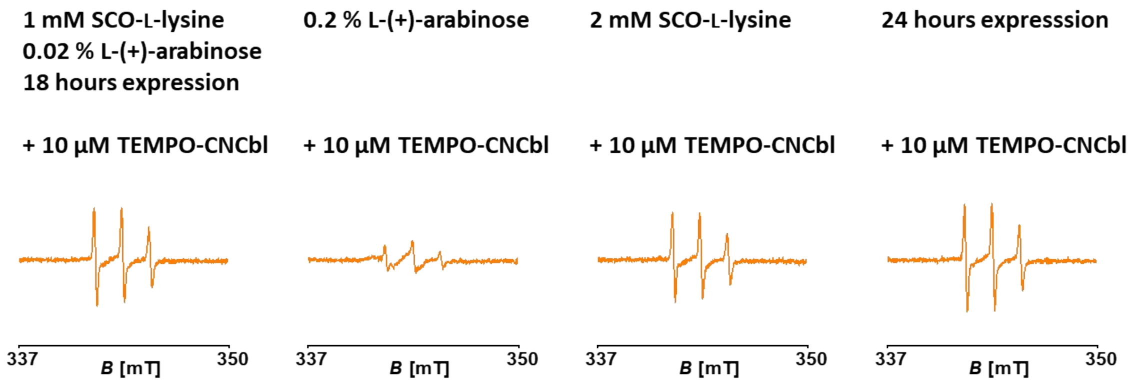

1 mL of *E. coli* at OD_{600} = 5 was pelleted, resuspended with MOPS buffer in a total volume of 25 µL and mixed with 10 µM TEMPO-CNCbl. Based on the expression conditions given above the left spectrum, one condition at a time was changed, as indicated above each spectrum. Bound TEMPO-CNCbl as observed by line broadening indicates improved expression of BtuB_F404⟶SCO only when increasing L-(+)-arabinose levels by a factor of 10.

Figure S7: Titration of BtuB_F404⟶SCO with TEMPO-CNCbl

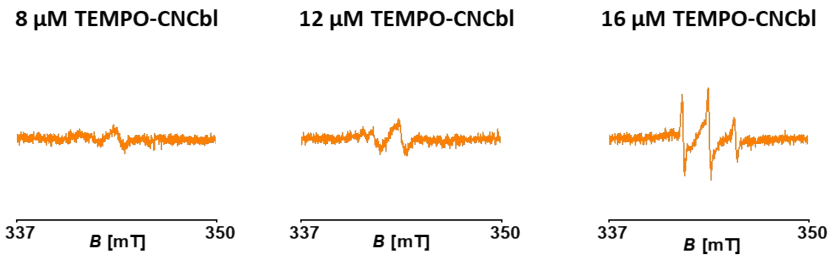

Semi-quantitative assessment of expression levels. 1 mL of *E. coli* at OD_{600} = 15 were pelleted, resuspended with MOPS buffer in a total volume of 25 µL and mixed with indicated amounts of TEMPO-CNCbl. When 16 µM TEMPO-CNCbl were added, certain fractions of unbound nitroxide were detected. Thus the concentration of BtuB was approximately 12 µM in this sample.

Figure S8: Generation of PaNDA rotamers

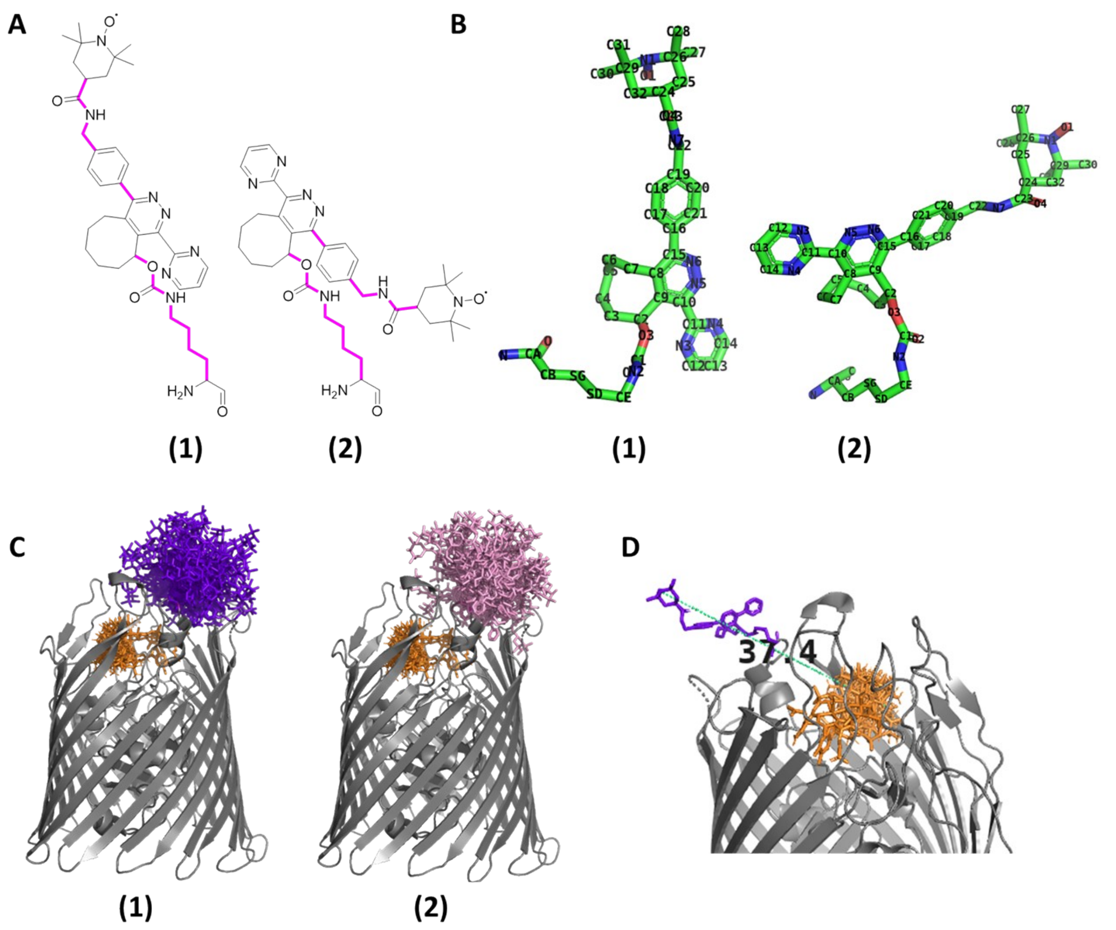

PaNDA rotamers were required to simulate spin labelling and DEER distance measurements.

A: ChemDraw structure of the PaNDA spin label attached to the ncAA SCO-L-lysine, considering the two possible regioisomers (1) and (2). The 13 bonds that form the rotation string are marked in purple.

B: PDB representation of the energy-minimized linker structures drawn in PyMOL, with all hydrogen atoms removed. All atom names were individually annotated conforming to the commonly used pattern. Carbon atoms are depicted in green, nitrogen atoms in blue and oxygen atoms in red.

C: Entirety of 71 rotamers of regioisomer (1) and 72 rotamers of regioisomer (2) attached to BtuB (PDB 1NQH)[11]. For this purpose, rotamers from the structures shown in **B** were generated with MtsslWizard as described in the Experimental Procedures section. The overlay of both rotamer populations is shown in **Figure 1C**, main text.

D: Part of the BtuB structure with one single exemplary rotamer structure of PaNDA regioisomer (1) (purple) attached and rotamers of TEMPO-CNCbl (orange). The distance between the PaNDA nitroxide radical and the TEMPO-CNCbl rotamers is indicated by a green dotted line and was determined with PyMOL as 3.74 nm (37.4 Å).

Figure S9: DEER data of PaNDA- and TEMPO-CNCbl-labelled sample *in situ*

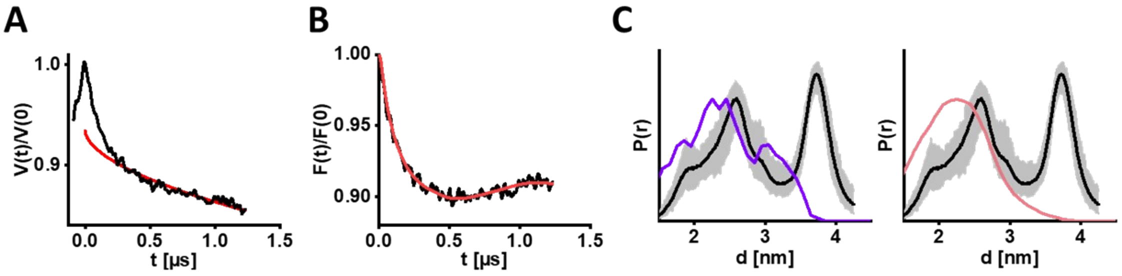

Evaluation of the DEER measurement of BtuB_F404⟶PaNDA additionally supplemented with TEMPO-CNCbl *in situ*. Data were processed using MATLAB R2019b, the DeerAnalysis2019[4] software and DEERNet.[5]

A: Primary DEER signal (black) and corresponding background fit (red).

B: Background-corrected form factor (black) and fit (red) acquired by DEERNet. Data is also shown in **Figure 4A**, main text. The modulation depth is 9.5 %.

C: Resulting distance distribution (black), validation acquired by DeerNet (grey shaded) and simulations (purple and light pink) based on two different regioisomers (**Figure S8**). The simulations were calculated by using MtsslWizard software[9] integrated into PyMOL (The PyMOL Molecular Graphics System, Version 2.4.1 Schrödinger, LLC.)[10] and the PDB crystal structure 1NQH[11]. The simulations were normalized to the height of the local maximum. The average of both simulations is shown in **Figure 4B**, main text.

Figure S10: PaNDA labelling in OM

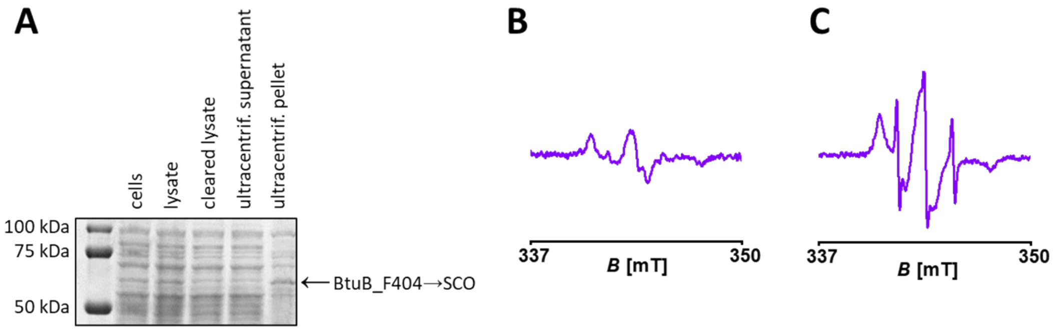

BtuB_F404⟶SCO was expressed in 400 mL RK5016 *E. coli*, before the isolated outer membranes (OM) were purified according to the protocol by Joseph et al.[3]

A: Analysis of BtuB levels by 10 % SDS PAGE during different steps of the outer membrane isolation. BtuB_F404⟶SCO was enriched in the membrane pellet resulting from ultracentrifugation (last lane).

B: Cw spectrum after PaNDA spin labelling in outer membranes and removal of excess label. The spectrum corresponds to approx. 25 µM PaNDA labelling.

C: Cw spectrum after PaNDA spin labelling and addition of approx. 35 µM TEMPO-CNCbl to the outer membranes. 20 % d_8-glycerol were added before the sample was frozen for DEER measurement (**Figure 4A**, main text, and **Figure S11**).

Figure S11: DEER data of PaNDA- and TEMPO-CNCbl-labelled sample in OM

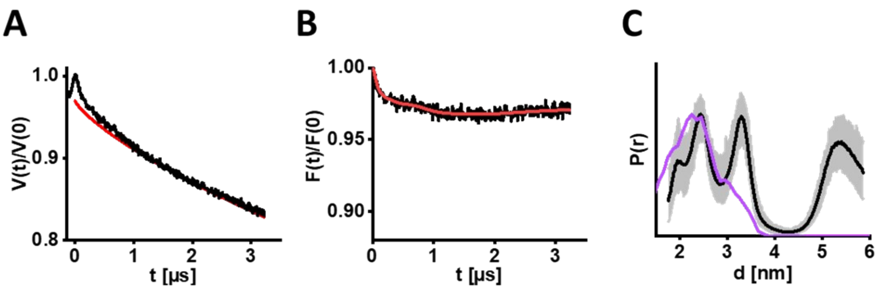

Evaluation of the DEER measurement of BtuB_F404⟶PaNDA additionally supplemented with TEMPO-CNCbl in isolated outer membranes (OM, for sample preparation see **Figure S10**). Data were processed using MATLAB R2019b, the DeerAnalysis2019[4] software and DEERNet.[5]

A: Primary DEER signal (black) and corresponding background fit (red).

B: Background-corrected form factor (black) and fit (red) acquired by DEERNet. Truncated data is also shown in **Figure 4A**, main text. The modulation depth is 3.0 %.

C: Resulting distance distribution (black), validation acquired by DeerNet (grey shaded) and simulation (purple; obtained and averaged as described in **Figure S9C**). The simulation was normalized to the height of the local maximum.

Figure S12: DEER data of PaNDA-labelled sample

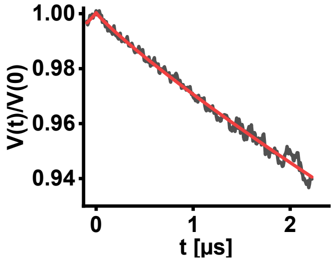

Evaluation of the DEER measurement of BtuB_F404⟶PaNDA labelled *in situ*. Primary DEER signal (black) and corresponding homogenous background fit (red) with dimensionality 2.7 are shown. The background start was set to 0 µs.

Figure S13: Viability of *E. coli*

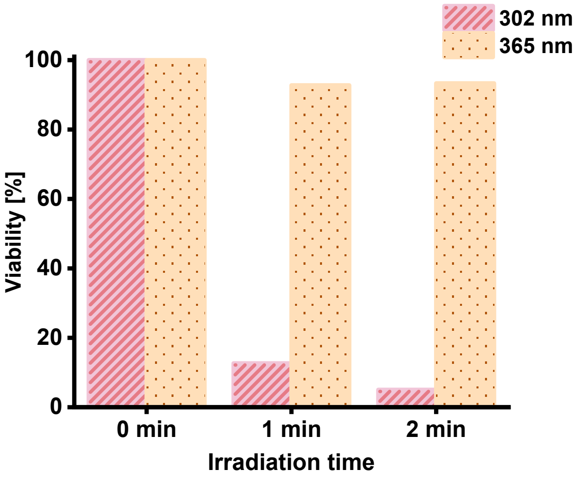

Viability of *E. coli* was assessed to ensure biocompatibility of the sample preparation. Viability was calculated as percentage of the control cells that were not irradiated (0 min) but have undergone labelling and washing. *E. coli* were irradiated at 302 nm (red bars) or 365 nm (yellow bars) for the indicated time period (1 or 2 min). Colony forming units per mL (CFU/mL) of the different samples were assessed by counting colonies on agar plates containing the appropriate antibiotics. *E. coli* viability was significantly reduced at shorter wavelength.

Figure S14: DEER data of MTSSL- and TEMPO-CNCbl-labelled sample *in situ*

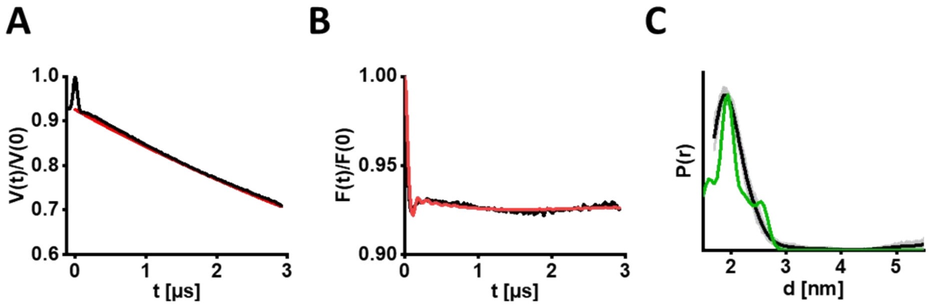

Evaluation of the DEER measurement of BtuB_F404→R1 (27 µM) additionally supplemented with TEMPO-CNCbl (27 µM) *in situ*. Data were processed using MATLAB R2019b, the DeerAnalysis2019[4] software and DEERNet.[5]

A: Primary DEER signal (black) and corresponding background fit (red).

B: Background-corrected form factor (black) and fit (red) acquired by DEERNet. The modulation depth is 7.4 %.

C: Resulting distance distribution (black), validation acquired by DeerNet (grey shaded) and simulation (green). The simulation was obtained by MM2018 software (http://www.epr.ethz.ch/software/index)[10] and the PDB crystal structure 1NQH.[11] The simulation was normalized to the maximum.

Figure S15: DEER data of MTSSL- and TEMPO-CNCbl-labelled sample in OM

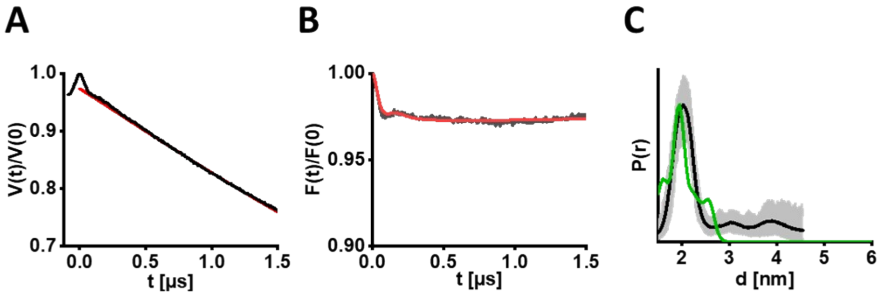

Evaluation of the DEER distance measurement of BtuB_F404⟶R1 additionally supplemented with TEMPO-CNCbl in isolated outer membranes (OM). Data were processed using MATLAB R2019b, the DeerAnalysis2019[4] software and DEERNet.[5]

A: Primary DEER signal (black) and corresponding background fit (red).

B: Background-corrected form factor (black) and fit (red) acquired by DEERNet. The modulation depth is 2.7 %.

C: Resulting distance distribution (black), validation acquired by DeerNet (grey shaded) and simulation (green; obtained as described in **Figure S14C**). The simulation was normalized to the maximum.

Supplementary References

1. K. Heller, B. Mann and R. Kadner, *J. Bacteriol.,* 1985, **161**, 896-903.
2. B. Joseph, A. Sikora, E. Bordignon, G. Jeschke, D. S. Cafiso and T. F. Prisner, *Angew. Chem.*, 2015, **127**, 6294-6297.
3. B. Joseph, E. A. Jaumann, A. Sikora, K. Barth, T. F. Prisner and D. S. Cafiso, *Nat. Protoc.*, 2019, **14**, 2344-2369.
4. G. Jeschke, V. Chechik, P. Ionita, A. Godt, H. Zimmermann, J. Banham, C. Timmel, D. Hilger and H. Jung, *Appl. Magn. Reson.*, 2006, **30**, 473-498.
5. S. G. Worswick, J. A. Spencer, G. Jeschke and I. Kuprov, *Sci. Adv.*, 2018, **4**, eaat5218.
6. B. Joseph, A. Sikora, E. Bordignon, G. Jeschke, D. S. Cafiso and T. F. Prisner, *Angew. Chem. Int. Ed.*, 2015, **54**, 6196-6199.
7. A. Scherer, S. Tischlik, S. Weickert, V. Wittmann and M. Drescher, *Magn. Reson.*, 2020, **1**, 59-74.
8. C. E. Tait and S. Stoll, *Phys. Chem. Chem. Phys.*, 2016, **18**, 18470-18485.
9. G. Hagelueken, R. Ward, J. H. Naismith and O. Schiemann, *Appl. Magn. Reson.*, 2012, **42**, 377-391.
10. Y. Polyhach, E. Bordignon and G. Jeschke, *Phys. Chem. Chem. Phys.*, 2011, **13**, 2356-2366.
11. D. P. Chimento, A. K. Mohanty, R. J. Kadner and M. C. Wiener, *Nat. Struct. Mol. Biol.,* 2003, **10**, 394-401.
